# Doctor, builder, soldier, lawyer, teacher, dancer, shopkeeper, vet: exploratory study of which eleven-year olds would like to become a doctor

**DOI:** 10.1186/s40359-015-0094-z

**Published:** 2015-11-04

**Authors:** I. C. McManus, Terry Ng-Knight, Lucy Riglin, Norah Frederickson, Katherine Shelton, Frances Rice

**Affiliations:** Research Department of Clinical, Educational and Health Psychology, University College London, Gower Street, London, WC1E 6BT UK; UCL Medical School, University College London, Gower Street, London, WC1E 6BT UK; School of Psychology, Cardiff University, Tower Building, 70 Park Place, Cardiff, CF10 3AT UK

**Keywords:** Medicine as a career, Children, Parents, 11-year olds, RIASEC, Occupational status

## Abstract

**Background:**

Very little is known about the extent to which eleven-year olds might consider a career in medicine. This exploratory study therefore asked children and their parents about medicine as a possible career, looking also at the relationship to a range of background measures.

**Methods:**

A longitudinal, three-wave, questionnaire study of students transferring from primary to secondary school (STARS), with data collection at primary school (wave 1; mean age 11.3 yrs), in the first months of secondary school (wave 2; mean age 11.7 yrs) and at the end of the first year of secondary school (wave 3; mean age 12.3 yrs). Parents/carers also completed questionnaires. Children were entering ten large comprehensive secondary schools in the south-east of England; 46.3 % were female, 15.6 % receiving free-school meals, 39.8 % were Black or Minority Ethnic and 28.8 % had a first language which was not English. Of 2287 children in the study, 1936 children (84.5 %) completed at least one questionnaire of the three waves (waves 1, 2 and 3). The main outcome measures were an open-ended question in each wave, “What job would you like to do when you grow up?”, and a more detailed questionnaire in wave 3 asking about 33 different jobs.

**Results:**

9.9 % of children spontaneously mentioned medicine as a career on at least one occasion. For the specific jobs, would-be doctors particularly preferred Hospital Medicine, followed by Surgery, General Practice and then Psychiatry. Would-be doctors were also more interested in careers such as Nurse, Archaeologist, Lawyer and Teacher, and less interested in careers such as Shopkeeper, Sportsperson, or Actor/dancer/singer/musician. Would-be doctors were less Neurotic, more Open to Experience, more Conscientious, and preferred higher prestige occupations. Those interested in medicine did not score more highly on Key Stage 2 attainment tests or Cognitive Abilities Test, did not have a higher family income or greater parental/carer education, and did not have more experience of illness or deaths among family and friends.

**Conclusions:**

An interest in a medical career, unlike high prestige jobs in general, is not associated with higher educational attainment or cognitive ability, and it is likely that only one in ten of the children interested in medical careers will have sufficient educational attainment at GCSE or A-level to be able to enter medical school.

## Background

The study of medical student selection usually begins when candidates submit their applications to a medical school, which in the UK is via the Universities and Colleges Admissions Service (UCAS), when most applicants are about 17 years old. Occasional studies have involved students at the age of 16 who are considering applying to medical school [1]. Prior to that age, though, all seems to be silence. The impetus for the present study was the first wave of a longitudinal survey of UK ten-year olds who were asked the simple open-ended question, “What job would you like to do when you grow up?”. Somewhat to our surprise, the answer ‘doctor’, or a related term such as ‘surgeon’ or ‘GP’, was given by nearly one in ten children (68/749; 9.1 %). Since such a high proportion implies potential for widening participation, we took the opportunity in two later waves to ask more detailed questions.

This study asks how many 11 year-olds consider medicine as a possible career, and asks how that interest relates to other career interests, and to demographic, life event, educational and personality measures. The study was in large part exploratory, but the questions we asked are informed by studies of career choice in adolescents and adults. Careers were described in terms of the system of vocational preferences developed by Holland [2] who showed that career choices can be mapped into six types (RIASEC: Realistic, Investigative, Artistic, Social, Enterprising and Conventional) [3]. Gottfredson has suggested that children’s career orientations change during development, younger children being primarily concerned with gender roles, while prestige becomes important during the ages 9 to 13, and specific fields of work, as characterised by RIASEC, become important from 14 onwards [4]. Whether the full RIASEC structure is present in children’s career choices is still unclear [5–7], although there is a suggestion that 11-year olds have a similar Social, Investigative and Realistic axis as adults (which Prediger called *People vs Things* [8]), but that Enterprising, Conventional and Artistic interests differ from those of adults [9]. We therefore classified jobs using both RIASEC structure and prestige levels. RIASEC types are also associated with personality dimensions [10], and adult occupations also relate to childhood personality measures [11], and so personality was also investigated.

The selection of medical students in the UK has been of increasing interest in recent years [12, 13], particularly given interests in widening access, both in general and particularly in medicine. A concern, inevitably, is that some children, despite an interest in careers such as medicine, subsequently lose that interest or do not have that interest encouraged and nurtured. It therefore makes sense to try and look at interests in medical careers at a much earlier stage, and the transition to secondary education makes a good baseline for that assessment. The Medical Schools Council report, in particular, discusses widening participation by outreach work within secondary and primary schools [13]. The GMC sponsored research [12] also comments that, “most [widening access] activities target secondary school pupils, most often those aged 14–16 years. *This is too late*.” (p.63, our emphasis). However neither report publishes any data on attitudes to medical careers in pupils below the age of about 14.

### A note on education in England

Compulsory education for children in the UK begins at age 5 (year 1), and there are differences between England, Scotland, Wales and Northern Ireland. The academic year runs from 1st September to 31st August. The present study took place in England, where primary education typically continues until year 6 (age 11). Children then move to secondary education in year 7, taking GCSE (General Certificate of Secondary Education) exams in year 11 (age 15) and for more academic students, AS-level and A-level examinations are taken in years 12 (age 16) and 13 (age 17). Students typically enter university at age 18. School progress is assessed at various ‘key stages’ (KS), KS1 for years 1–2, KS2 for years 3–6, KS3 for years 7–9, KS4 for years 10–11 and KS5 for years 12–13. Assessments vary in type, sometimes being teacher assessments, sometimes being formal assessments, and sometimes being based on GCSE, AS and A-level examinations. In addition many schools and local authorities use tests such as the Cognitive Abilities Tests both to stream pupils, and also to allocate students to schools (where they are used to ensure diversity of ability ranges). Most state schools in England are comprehensive (and all of the schools in the present study are comprehensive), but there are also selective schools, both in the state and the private sector. For further information see https://www.gov.uk/government/collections/national-curriculum-assessments-test-frameworks.

## Methods

The School Transition and Adjustment Research Study (STARS) (http://www.ucl.ac.uk/stars) is a longitudinal study of a large group of 11-year olds in their last year at primary schools (Year 6) in the south-east of England. The primary interest of STARS is in transitions, and how children respond to them, but the nature of the study means that there is potential for studying many other questions as well. The children were followed during the transition to secondary education, until the end of the first year at secondary school (Year 7) [14, 15]. The children entered ten large comprehensive (non-selective) schools in south-east England, an average of 205 children entering each school (range 120 to 290; SD 67).

Questionnaires were given to Children (C), Parents/Carers (P) and Teachers (T), C1, P1 and T1 being completed at the end of the final term of primary school, C2, P2 and T2 in the first term at secondary school, and C3, P3 and T3 at the end of Year 7 (May 2013) (see Table 1). Questionnaires were extensive, and only some items are described here. In particular there were measures of the Big Five personality scales [16], 21 items from the BFI (http://www.ocf.berkeley.edu/~johnlab/bfi.php) being completed by the parent/carer in P1. Schools provided background demographic measures, as well as measures of attainment at Key Stage 2 (KS2) and, where known, scores on the Cognitive Abilities Test (CAT) which is widely used in UK schools for selection, banding or prediction of likely attainment [17, 18]. Seven of the ten schools also provided teacher assessments of Key Stage 3 (KS3) attainment for National Curriculum levels at the end of year 7, in English, Maths and Science. Scores vary from 2c, through 2b, 2a, 3c, 3b, 3a, etc., to 7c and 7b, and are scored from 1 to 17, with an overall score calculated as an average (see https://www.gov.uk/government/collections/national-curriculum-assessments-test-frameworks).

**Table 1:** “What job would you like to do when you grow up?” (child) or “what job would you like your child to do when they grow up?” (parent/carer)

	Mean date of completion [mean age in years of child; SD]		“Medicine” mentioned by:	
	Child	Parent/Carer	Child	Parent/Carer
Wave 1	28th May 2012 [11.25;.293)	26th May 2012	9.8 % (68/695)	7.5 % (56/745)
Wave 2	17th Nov 2012 (11.72;.292)	28th Oct 2012	12.8 % (66/515)	7.4 % (40/544)
Wave 3	15th Jun 2013 (12.29; .295)	n/a	8.7 % (111/1272)	n/a
Mentioned at any wave			9.8 % (154/1564)	9.7 % (86/887)

Children were born between 1st September 2000 and 31st August 2001, and mean ages are given for children at various stages of the study. For parents the median date of completion of the questionnaire is given

### Career measures

In C1, C2 and C3 children were asked, “What job would you like to do when you grow up?”, and in P1 and P2, parents/carers were asked, “What job would you like your child to do when they grow up?”. Responses were free text, were transcribed into the computer, and subsequently coded by ICM into various categories (see below). In C3, children were also asked a more detailed question, “Here is a list of different jobs that people do. Say how much you might like to do each one by ticking one of the smiley faces next to it”, with answers on a three-point scale (“☺: Would like it a lot” / “: Not sure” / “☹: Wouldn’t like it at all”). Thirty-three different jobs were presented, in alphabetical order. Four jobs were for different aspects of medicine (Surgeon, Hospital Doctor, GP and Psychiatrist). The remaining 29 jobs (see Table 2) broadly sampled the six RIASEC categories. Jobs were also sampled from high and low status occupations.

**Table 2:** Liking for different jobs for those who spontaneously mentioned (*N* = 129) or did not mention (*N* = 1170) medicine on any of the open-ended questions^a^

RIASEC	SOC 2000		☺ Would like this job		☹ Wouldn’t like this job		Kendall’s τ_c_ , P
			Medicine mentioned	Medicine not mentioned	Medicine mentioned	Medicine not mentioned	
Medical specialties							
ISr	2	**Hospital doctor**	**84.5 % (109)**	**20.4 % (239)**	**3.9 % (5)**	**54.4 % (636)**	**.248, ** ***p*** ** < .001**
RIS	2	**Surgeon**	**46.5 % (60)**	**11.8 % (138)**	**32.6 % (42)**	**67.9 % (795)**	**.151, ** ***p*** ** < .001**
ISr	2	**GP (General practitioner)**	**36.4 % (47)**	**6.8 % (80)**	**37.2 % (48)**	**61.4 % (718)**	**.121, ** ***p*** ** < .001**
ISa	2	**Psychiatrist**	**21.7 % (28)**	**7.9 % (93)**	**46.5 % (60)**	**64.4 % (753)**	**.076, ** ***p*** ** < .001**
Other jobs							
SIr	3	**Nurse**	**41.1 % (53)**	**12.9 % (151)**	**33.3 % (43)**	**65.4 % (765)**	**.135, ** ***p*** ** < .001**
Ir	2	**Scientist**	**52.7 % (68)**	**23.1 % (270)**	**24.0 % (31)**	**49.6 % (580)**	**.124, ** ***p*** ** < .001**
Eia	2	**Lawyer**	**48.1 % (62)**	**26.0 % (304)**	**22.5 % (29)**	**47.9 % (560)**	**.108, ** ***p*** ** < .001**
E	2	**Businessperson**	**50.4 % (65)**	**30.1 % (352)**	**20.9 % (27)**	**37.0 % (433)**	**.086, ** ***p*** ** < .001**
CE	2	**Accountant**	**23.3 % (30)**	**11.3 % (132)**	**31.8 % (41)**	**45.6 % (534)**	**.067, ** ***p*** ** < .001**
IRa	2	**Archaeologist**	**24.8 % (32)**	**14.7 % (172)**	**41.1 % (53)**	**55.6 % (651)**	**.060, ** ***p*** ** = .001**
Aei	3	**Journalist**	**19.4 % (25)**	**17.9 % (209)**	**35.7 % (46)**	**54.2 % (634)**	**.057, ** ***p*** ** = .001**
IRc	2	**Engineer**	**36.4 % (47)**	**21.7 % (254)**	**42.6 % (55)**	**52.9 % (619)**	**.054,** ***p*** ** = .004**
Sa	2	**Teacher**	**22.5 % (29)**	**21.5 % (251)**	**32.6 % (42)**	**47.2 % (552)**	**.043, ** ***p*** ** = .011**
Rci	5	Mechanic	19.4 % (25)	13.4 % (157)	55.8 % (72)	63.7 % (745)	.032, *p* = .065
IC	2	Computer programmer	31.8 % (41)	25.8 % (302)	43.4 % (56)	49.0 % (573)	.026, *p* = .156
ESc	6	Air steward/Flight attendant	10.9 % (14)	10.2 % (119)	53.5 % (69)	59.7 % (698)	.021, *p* = .220
RCi	3	Airline pilot	19.4 % (25)	17.4 % (204)	54.3 % (70)	59.4 % (695)	.018, *p* = .294
RE	3	Police officer	31.0 % (40)	24.7 % (289)	45.0 % (58)	45.5 % (532)	.014, *p* = .462
IR	2	Vet	18.6 % (24)	17.8 % (208)	52.7 % (68)	56.8 % (665)	.013, *p* = .435
Si	2	Social worker	14.0 % (18)	10.6 % (124)	62.8 % (81)	63.6 % (744)	.007, *p* = .680
EC	7	Telephone salesperson	2.3 % (3)	1.8 % (21)	85.3 % (110)	86.6 % (1016)	.006, *p* = .625
AR	3	Artist	27.1 % (35)	29.2 % (342)	39.5 % (51)	41.3 % (483)	.000, *p* = .999
Ce	4	Secretary	9.3 % (12)	8.5 % (99)	62.8 % (81)	62.1 % (727)	-.001, *p* = .952
Rc	5	Gardener	3.1 % (4)	3.8 % (45)	79.8 % (103)	77.7 % (909)	-.008, *p* = .550
Ae	2	Videogame designer	34.9 % (45)	36.1 % (422)	48.8 % (63)	42.5 % (497)	-.017, *p* = .351
(RE)	(3)	Soldier/Sailor/RAF	17.1 % (22)	18.4 % (215)	65.1 % (84)	58.7 % (687)	-.021, *p* = .216
ECs	2	**Shopkeeper**	**7.0 % (9)**	**8.4 % (98)**	**76.7 % (99)**	**68.5 % (801)**	**-.029, ** ***p*** ** = .050**
AE	3	Designer	39.5 % (51)	45.2 % (529)	30.2 % (39)	23.1 % (270)	-.030, *p* = .107
Reis	6	**Hairdresser/barber**	**10.9 % (14)**	**14.5 % (170)**	**71.3 % (92)**	**60.1 % (703)**	**-.040, ** ***p*** ** = .013**
Re	3	**Sportsperson**	**34.1 % (44)**	**43.9 % (514)**	**42.6 % (55)**	**34.4 % (402)**	**-.040,** ***p*** ** = .026**
EA	3	**Film producer/director**	**34.1 % (44)**	**42.1 % (492)**	**34.1 % (44)**	**24.5 % (287)**	**-.041, ** ***p*** ** = .026**
R	5	**Builder/decorator**	**15.5 % (20)**	**18.3 % (214)**	**62.8 % (81)**	**49.7 % (582)**	**-.043, ** ***p*** ** = .014**
Ae	2	**Actor/dancer/singer/musician**	**33.3 % (43)**	**46.3 % (542)**	**35.7 % (46)**	**26.5 % (310)**	**-.052, ** ***p*** ** = .004**

^a^Jobs were rated on a three-point scale (“☺: Would like it a lot” / : Not sure” / “☹: Wouldn’t like it at all”), but only percentages of the first and last are presented. Kendall’s tau (τ_c_) is calculated using all three groups, and significant associations with p < .05 are shown in bold. Jobs were presented in alphabetical order in the questionnaire, but here are sorted by Kendall’s tau. RIASEC codes are shown in size order, those greater than 5 in upper case and those between 4 and 5 in lower case. SOC2000 codes for occupational prestige are from 1 to 7 (but no 1 s were included). Note that Ns are slightly lower than in Table 1 due to not all children who had mentioned medicine answering the C3 questionnaire

Scores on the six RIASEC scales for each job were obtained from the *Occupation_Data* and *Interests* files of O*NET 18.0 (http://www.onetcenter.org/db_releases.html), and are in the range 7 (high) to 1 (low). Prestige for each job was based on the Standard Occupational Classification 2000 (SOC2000) of the UK Office for National Statistics which varies from level 2 (Professional Occupations; *n* = 16), 3 (*n* = 10), 4 (*n* = 1), 5 (*n* = 3), 6 (*n* = 2) and 7 (Sales and Customer Service Occupations; *n* = 1). Scores for RIASEC and prestige for each job are indicated in Table 2. For each child an average score on the six RIASEC scales and the prestige scale was calculated as the mean weighted preferences for the 29 non-medical jobs. For ease of interpretation, SOC2000 scores were reversed so that high scores indicate high prestige. From the RIASEC scores we also calculated Prediger’s [8] scales of *People vs Things* (2S + E-C-2R-I + A) and *Data vs Ideas* (1.7E + 1.7C −1.7I – 1.7A).

A checklist of 34 positive and negative life events in the previous year was provided in waves 1 and 3 for children and wave 1 for parents/carers, and we calculated composite scores for any report of death of family and friends, or of serious illness or injury among family, friends or the child themself.

Ethical approval for the study was obtained from the University College London Research Ethics Committee (ID number = 1522/01). Informed consent (parents/carers) and assent (children) was obtained from all participants.

Statistical analysis used *SPSS 22.0*. Differences between groups were compared using chi-square, Kendall’s tau and t-tests as appropriate. Multivariate analysis used logistic regression with missing values substituted using the EM (expectation-maximisation) algorithm in *SPSS*.

## Results

Of the children in the study, 46.3 % (1058/2287) were female, 15.6 % (274/1762) received free school meals, 39.8 % (685/1723) were Black or Minority Ethnic (BME: Asian 389, Black 124, Mixed ethnicity 128, Other 44), and 28.8 % (507/1763) had a first language which was not English (with differing denominators reflecting different response rates on the various questionnaires). Mean gross family income was about £37 K per annum, and 29 % of children had at least one parent/carer with a degree. The percentage of non-white students at each school varied from 9.9 % to 92.1 % (mean = 64.7 %; median = 82.7 %; SD = 32.0 %). For state-maintained secondary schools in England in 2001, the average percentage of non-white students was 13 %, but there was a wide variation with many more non-white pupils in the South East of England [19].

In all three waves, children were asked the open-ended question, “What job would you like to do when you grow up?”. Of 749 children in wave 1, 695 (92.8 %) named a job, the remainder leaving the answer blank or saying “Don’t Know”. The total range was wide, including, for instance, “comic book artist”, “hairdresser or something to with animals”, “Lego designer”, “mathematiciam and Part-time magician”, “run my own Catery”, “childminder like my mum” and “bounty hunter” (all spelling as in original). A number of common themes emerged however, and for wave 1 thirteen categories emerged which accounted for over half of the jobs named; medicine was at the top (9.8 %, 68/695). , followed by Actor/Dancer/Singer (59), Sportsperson (57), Teacher (51), Police Officer (31), Lawyer (26), Vet (24), Scientist (19), Engineer (17), Pilot (15), Computers/Videogames (6), Nurse (5) and Childcare/Nursery (4), together accounting for 382 (55.0 %) of named jobs. Medicine was coded broadly, and responses varied from single, focussed responses, both generic (‘A doctor’; ‘Docter’), and specific (‘Doctor GP’; ‘Brain/Heart Surgeon”; ‘Brian surgeon’; ‘Pathologist’), to other cases where medicine was listed with other possible careers (‘Doctor, singer’; ‘I would like to be a pedetriton, a normal doctor or even a teacher who works in school’; ‘Actor/singer/doctor/soldier/spy/footballer’); all were coded as indicating some interest in medicine. Open-ended responses in waves 2 and 3 showed the same pattern as in wave 1.

About 10 % of children (and also parents/carers) spontaneously mentioned medicine or a related term as a job they would like to do (or like their child to do), in at least one of the waves (see Table 1). For children, mentioning medicine showed consistency across waves 1 and 2 (phi = .517, *p* < .001), 1 and 3 (phi = .511, *p* < .001), and waves 2 and 3 (phi = .432, *p* < .001), as also did parents mentioning medicine in waves 1 and 2 (phi = .342, *p* < .001). There was also a correlation between the child and the parent/carer mentioning medicine (phi = .554, *p* < .001). Further analyses consider those 9.8 % of children or those 9.7 % of parents/carers who mentioned medicine on at least one wave.

In wave 3, as well as being asked the open-ended question about jobs, children also rated their interest in 33 different jobs using a three-point scale (see Table 2). Career preferences are often negative [20], people being more certain what they don’t want to do than what they do want to do, and in these data children made a mean of 6.7 positive choices (SD 4.9) but 17.4 (SD 7.4) negative choices (t = 35.5, 1437 df, *p* < .001).

Of the four medical categories the highest popularity was for Hospital Doctor with 24.3 % (350/1438) saying they would like to do it, compared with 14.3 % (206/1438) for Surgeon, 9.2 % for GP (132/1438) and 8.9 % (128/1438) for Psychiatrist. Table 2 shows how preferences for each specific career related to a child spontaneously mentioning medicine in the open-ended questions. ‘Medicine’ was mostly strongly related to ‘Hospital doctor’, then to ‘Surgeon’, ‘GP’ and finally ‘Psychiatrist’, suggesting that the archetypical perception of medicine is as a hospital doctor. Those spontaneously choosing medicine also tended to choose Nurse, Scientist and other realist-investigative careers such as Engineer and Archaeologist, but also Teacher, Journalist, Lawyer, Businessperson and Accountant. Those choosing medicine did not want to be a Shopkeeper, Hairdresser/Barber, Sportsperson, Film Producer/Director, Builder/Decorator or Actor/dancer/singer/musician. There was no association of medicine with jobs such as Vet or Social Worker.

Table 3 shows the association of spontaneously mentioning medicine with a range of background measures. Amongst the demographic variables there are strong associations with being Black or Minority Ethnic, with not having English as a first language, and being female, but not with free school meals, family income or parental/carer education level. Forward-entry logistic regression found that both BME and not having English as a first language were both independent predictors of wanting to study medicine. School attainment measures showed no differences either at KS2, in the cognitive ability measures, or in the measures at KS3, between those who did and did not want to become doctors. There were however differences in personality, those wanting to be doctors being more conscientious, more open to experience and less neurotic. Those wanting to be doctors also showed differences in the other (non-medical) job types that they preferred, being more interested in Investigative, Social, and Enterprising jobs, and less interested in Artistic or Conventional jobs. In addition, they were more likely to choose higher prestige jobs over lower prestige jobs.

**Table 3:** Association of spontaneously mentioning medicine with demographic, educational, personality and job type measures

	Mentioned medicine	Did not mention medicine	Significance
	Mean (SD;N) or % (N)	Mean (SD;N) or % (N)	
Demographic measures			
** Sex (% Female)**	**60.4 % (154)**	**47.2 % (1410)**	**Phi = .079, X** ^**2**^ ** = 9.72, 1df,** ***p*** ** = .002**
** Ethnicity (% BME)**	**74.8 % (107)**	**35.9 % (1213)**	**Phi = .244; X** ^**2**^ ** = 80.5, 1df,** ***p*** ** < .001**
** English not first language**	**60.7 % (145)**	**26.2 % (325)**	**Phi = .231 , X** ^**2**^ ** = 73.7, 1df,** ***p*** ** < .001**
Free school meals	14.5 % (145)	14.6 % (1239)	Phi = −.001; X^2^ = .002, 1df, *p* = .968
Family income (£000)	33.1 (22.6; 59)	37.9 (19.8; 510)	t = −1.70, 567 df, *p* = .091
Parental education level	2.43 (1.09; 63)	2.20 (1.17; 570)	t = 1.47, 631 df, *p* = .142
Life events			
Death of parent, brother, sister, grandparent or close friend	20.8 % (154)	25.5 % (1410)	Phi = −.032, X^2^ = 1.62, 1df, *p* = .203
Serious illness or injury in self, family or close friend	24.7 % (154)	28.9 % (1410)	Phi = −.028, X^2^ = 1.24, 1df, *p* = .266
School attainment			
KS2 English	4.35 (.68; 139)	4.26 (.69; 1197)	t = 1.36, 1334 df, *p* = .175
KS2 maths	4.41 (.76; 139)	4.30 (.76; 1203)	t = 1.72, 1340 df, *p* = .273
Cognitive ability test; verbal	101.7 (13.0; 96)	103.2 (12.1; 869)	t = −1.17, 963 df, *p* = .244
Cognitive ability test; non-verbal	104.6 (14.7; 96)	102.3 (13.8; 869)	t = 1.53, 963 df, *p* = .127
Cognitive ability test; quantitative	103.9 (14.4; 96)	102.2 (13.5; 869)	t = 1.20, 963 df, *p* = .231
KS3 (year 7) English	10.70 (2.36; 128)	10.86 (2.53; 1132)	t = −.675 , 1258 df, *p* = .500.
KS3 (year 7) Maths	11.94 (2.75; 128)	11.42 (3.01; 1134)	t = 1.89 , 1260 df, *p* = ..060
KS3 (year 7) Science	11.43 (2.13; 128)	11.07 (2.15; 1135)	t = 1.84 , 1261 df, *p* = 067.
KS3 (year 7) average	11.36 (2.05; 128)	11.11 (2.28;1135 )	t = 1.17, 1261 df, *p* = .241
Personality			
** Neuroticism**	**2.63 (1.13; 76)**	**3.02 (1.07; 613)**	**t = −3.04, 687 df,** ***p*** ** = .002**
Extraversion	3.74 (.77; 74)	3.70 (.85; 613)	t = .448, 687 df, *p* = .654
** Openness to experience**	**4.37 (.64; 76)**	**4.14 (.73; 614)**	**t = .2.68, 688 df, ** ***p*** ** = .007**
Agreeableness	4.45 (.68; 76)	4.30 (.76; 615)	t = 1.62, 689 df, *p* = .106
** Conscientiousness**	**3.91 (.75; 76)**	**3.91 (.75; 613)**	**t = 2.81, 687 df,** ***p*** ** = .005**
Job preferences			
** Preference for Realistic jobs (R)**	**3.85 (.17; 129)**	**3.91 (.20; 1170)**	**t = −3.00, 1297 df,** ***p*** ** = .003**
**Preference for Investigative jobs (I)**	**3.43 (.19; 129)**	**3.28 (.20; 1170)**	**t = 8.52, 1297 df,** ***p*** ** < .001**
** Preference for Artistic jobs (A)**	**3.32 (.21; 129)**	**3.38 (.24; 1170)**	**t = −2.73, 1297 df,** ***p*** ** = .006**
Preference for Social jobs (S)	3.07 (.17; 129)	3.03 (.17; 1170)	t = 1.93, 1297 df, *p* = .053
**Preference for Enterprising jobs (E)**	**4.17 (.13; 129)**	**4.21 (.14; 1170)**	**t = −2.73, 1297 df, ** ***p*** ** = .006**
**Preference for Conventional jobs (C)**	**3.71 (.14; 129)**	**3.67 (.15; 1170)**	**t = 2.53, 1297 df,** ***p*** ** = .011**
People (SAE) rather than Things (RIC)	−1.22 (.81; 129)	−1.10 (1.02; 1170)	t = −1.24, 1297 df, *p* = .216
**Data (CE) rather than Ideas (IA)**	**1.92 (.68; 129)**	**2.08 (.73; 1170)**	**t = 2.44, 1297 df,** ***p*** ** = .015**
**Preference for high prestige jobs**	**1.30 (.721; 121)**	**.249 (.856; 1124)**	**t = 13.02, 1243 df, ** ***p*** ** < .001**

The 25 variables in Table 3 were explored further using logistic regression, with missing values handled using the EM algorithm, and an alpha level of .001 to control for multiple testing. Five variables were statistically significant, an interest in medicine being predicted, in order of significance, by being more interested in high prestige jobs other than medicine, being non-white, being more open to experience, not being interested in Artistic careers, and being female, all with *p* < .001. Since the effects of ethnicity and sex were of particular interest, the seven interactions of sex and ethnicity with the other variables were tested but none reached a Bonferroni corrected significance level of 0.05/7.

## Discussion

About one in ten eleven-year old children, when asked what job they would like to do when they grow up, spontaneously answer ‘doctor’, and they do so reliably across three separate occasions over a year. About one in ten of the children’s parents/carers also spontaneously say that they would like their child to be a doctor, and that does not correlate with parental/carer levels of education. Although we know of no previous studies asking eleven-year old children whether they would like to become a doctor, the *BMJ* in 1946 did describe the results of an opinion poll which asked adults, “If you had a son [*sic*] starting out in life, what kind of work would you like him to take up?”, to which 7.5 % of respondents said medicine, a figure similar to the 9.7 % found here [21].

Children wanting to be a doctor are more likely to be female and to come from ethnic minorities (and also not to have English as a first language), and in that sense they resemble medical students themselves, who are also more likely than the population to be female or from ethnic minorities. Compared with other schoolchildren, would-be doctors show different patterns of job interests, and are less interested in Holland’s Artistic and Conventional groups of jobs and more interested in high prestige jobs (which more often are Investigative or Social). Those thinking of studying medicine are more open to experience, more conscientious and less neurotic than other children, the latter two characteristics, along with school attainment, being associated with an interest in higher prestige jobs in general, rather than medicine as such. Although there is some evidence that medical student specialty preferences are related to personal experience of illness [22, 23], there was no evidence that those wanting to be a doctor had more experience of illness or death.

Surprisingly, perhaps, given that medical students have some of the highest A-level grade attainments of any university applicants, there was no significant correlation of a preference for medicine as a career with school attainment, either with Key Stage 2 testing in year 6, or with the Cognitive Abilities Test (CAT) [17, 18]. CAT scores correlate very highly with GCSE attainment [24], and GCSEs in turn correlate highly with A-level grades [25]. A minimum attainment at GCSE and A-levels for having a chance of entering medical school is eight A grades in the best 8 GCSEs, and three A grades at A-level, including an A in Chemistry. To achieve those GCSE grades (416 points, or an A at A-level Chemistry, would require about 121 or 117 points at CAT^1^. CAT scores of 117 and 131 would give 25 and 50 % chances of an A grade in A-level Chemistry. Considering just the mid-point of those latter estimates (124), only 9.6 % (9/96) of those wanting to study medicine had a CAT score on the quantitative scale of at least 124, and therefore had any reasonable chance of actually doing so given medical school entry criteria. The remaining 90 % will probably therefore be disappointed in their aspirations. Amongst those not mentioning medicine at age 11, 5.0 % (43/869) had quantitative CAT scores of 124 or more, and might of course at a later date choose to study medicine.

An interesting question is therefore why so many children (and their parents/carers) have what, it is sad to say, are probably unrealistic expectations. Although wanting to be a doctor does not correlate with academic attainment, children in the sample who wished to have higher prestige jobs in general have significantly higher KS2 and CAT scores, and their parents/carers have higher levels of education, suggesting that children mostly do realise that some jobs require higher attainment levels. Medicine though does not show that pattern, despite medical schools asking for attainment in the top 1 or 2 % of the population, and academic attainment correlating with achievement at medical school [26]. One explanation may be that many people have experience of visiting doctors and hospitals (unlikely visiting lawyers or engineers), but few realise the technical and scientific underpinnings of the job, perhaps instead seeing what seems mainly to be a caring, intuitive task which could be carried out by many people.

An interesting question concerns the type of medicine in which children are interested. The detailed questions in C3, shown in Table 2, show clearly that it is hospital medicine and then surgery which are of the greatest interest, with a much lower proportion having an interest in General Practice. At a time when there is a national shortage of doctors interested in becoming GPs, that suggests that the lack of interest may have deeper origins.

Finally, is it reasonable to have made a decision to study medicine at the age of eleven? In the 1991 Cohort Study [27], one of us [ICM] asked entrants to medical school at what age they had first decided to study medicine and at what age they had definitely decided to study medicine. 44.7 % (1323/2959) had first considered studying medicine by the age of eleven, but only 5.5 % (164/2980) said they had definitely decided by that age. Of interest is that the gender and ethnic balance of those interested in medicine in our sample closely reflect the current medical student profile, suggesting that the pool from which medical students are drawn may be largely defined by 11 years? We will therefore be following the STARS cohort with great interest to find who actually does go on to study medicine.

### Study limitations

This is a single study, although it is longitudinal and of a reasonable sample size, looking at entire populations within a number of schools. Care should therefore be taken in generalizing from its results.

## Conclusions

About one in ten eleven-year olds spontaneously mentions medicine as a possible career, which has implications for widening diversity in medicine. Female students and minority ethnic students were more likely to mention medicine, and would-be doctors differed in personality and other careers which would be considered. There was no relationship between considering medicine as a career and performance on cognitive ability tests or school attainment measures.

## Footnotes

1 For general information on the Cognitive Abilities Test see http://www.gl-assessment.co.uk/products/cat-cognitive-abilities-test/performance-indicators. Detailed information on CAT and GCSEs is available at http://www.gl-assessment.co.uk/sites/gl/files/images/Files/GCSE_Technical_Information.pdf and a spreadsheet is also available at http://www.docstoc.com/docs/163828315/GCSE-Results-2012. A detailed Excel spreadsheet on the prediction of A-levels can be found at http://www.gl-assessment.co.uk/sites/gl/files/images/Files/For%20Website%20A%20level%20indicators%20published%20V2.xlsx.
